# ﻿*Aphyllorchisperiactinantha* (Orchidaceae, Neottieae), a new mycoheterotrophic species from peninsular Thailand

**DOI:** 10.3897/phytokeys.215.91458

**Published:** 2022-12-19

**Authors:** Amonrat Chantanaorrapint, Sahut Chantanaorrapint

**Affiliations:** 1 Faculty of Natural Resources, Prince of Songkla University, Hat Yai, Songkhla 90110, Thailand Prince of Songkla University Songkhla Thailand; 2 PSU Herbarium, Division of Biological Science, Faculty of Science, Prince of Songkla University, Hat Yai, Songkhla, 90110, Thailand Prince of Songkla University Songkhla Thailand

**Keywords:** achlorophyllous orchid, dipterocarp forest, Epidendroideae, peloric flower, Thai-Malay Peninsula, Ton Nga Chang Wildlife Sanctuary

## Abstract

A new orchid species from southern Thailand, *Aphyllorchisperiactinantha*, is described and illustrated. The novelty is characterized by the subactinomophic flowers, the concave labellum, not divided into hypochile and epichile, the reduced staminodes, the shallowly bilobed stigma and the semicircular rostellum. A key to the species of *Aphyllorchis* in Thailand is updated.

## ﻿Introduction

*Aphyllorchis* Blume is one of the largest mycoheterotrophic orchid genera with 19 currently accepted species (POWO 2022), mainly distributed in tropical Asia and the Himalayas, extending to Japan and Australia (Pridgeon et al. 2005; Suetsugu et al. 2018; Qin et al. 2021). Members of this genus are leafless and achlorophyllous herbs with erect and unbranched stems, racemose, many-flowered-inflorescences, petals similar to sepals but shorter and narrower, and a labellum usually divided into hypochile and epichile (Pridgeon et al. 2005; Hsieh et al. 2013; Tian et al. 2013; Suetsugu et al. 2018; Qin et al. 2021). In Thailand, four species have been recorded: *A.caudata* Rolfe ex Downie, *A.evrardii* Gagnep., *A.montana* Rchb.f. and *A.pallida* Blume (Downie 1925; Seidenfaden 1978; Roy et al. 2009; Pedersen 2014).

In November 2015, during an orchid survey in Ton Nga Chang Wildlife Sanctuary (TNCWS), Songkhla province, southern Thailand, an immature inflorescence of an unknown achlorophyllous orchid was observed. However, due to the immature flowers, it could not be accurately determined. Later, four further botanical surveys to TNCWS and Ban Yang Ko community forest resulted in four more collections of this unknown orchid. After careful examination, these specimens were identified as belonging to *Aphyllorchis*, however, they differed from all the other known species of the genus in Thailand. These specimens resemble *A.anomala* Dockr. from Australia, A.montanaRchb.fvar.rotundatipetala (C.S.Leou, S.K.Yu & C.T.Lee) T.P.Lin from Taiwan, *A.simplex* Tang & F.T.Wang from China and Vietnam, and *A.striata* (Ridl.) Schltr. from Peninsular Malaysia and Borneo in having subactinomophic (peloric) flowers and a labellum not divided into hypochile and epichile as in most species of *Aphyllorchis*. Following a detailed comparison with closely related taxa, we here describe and illustrate these specimens as a new species.

## ﻿Materials and methods

**Figure 1.**
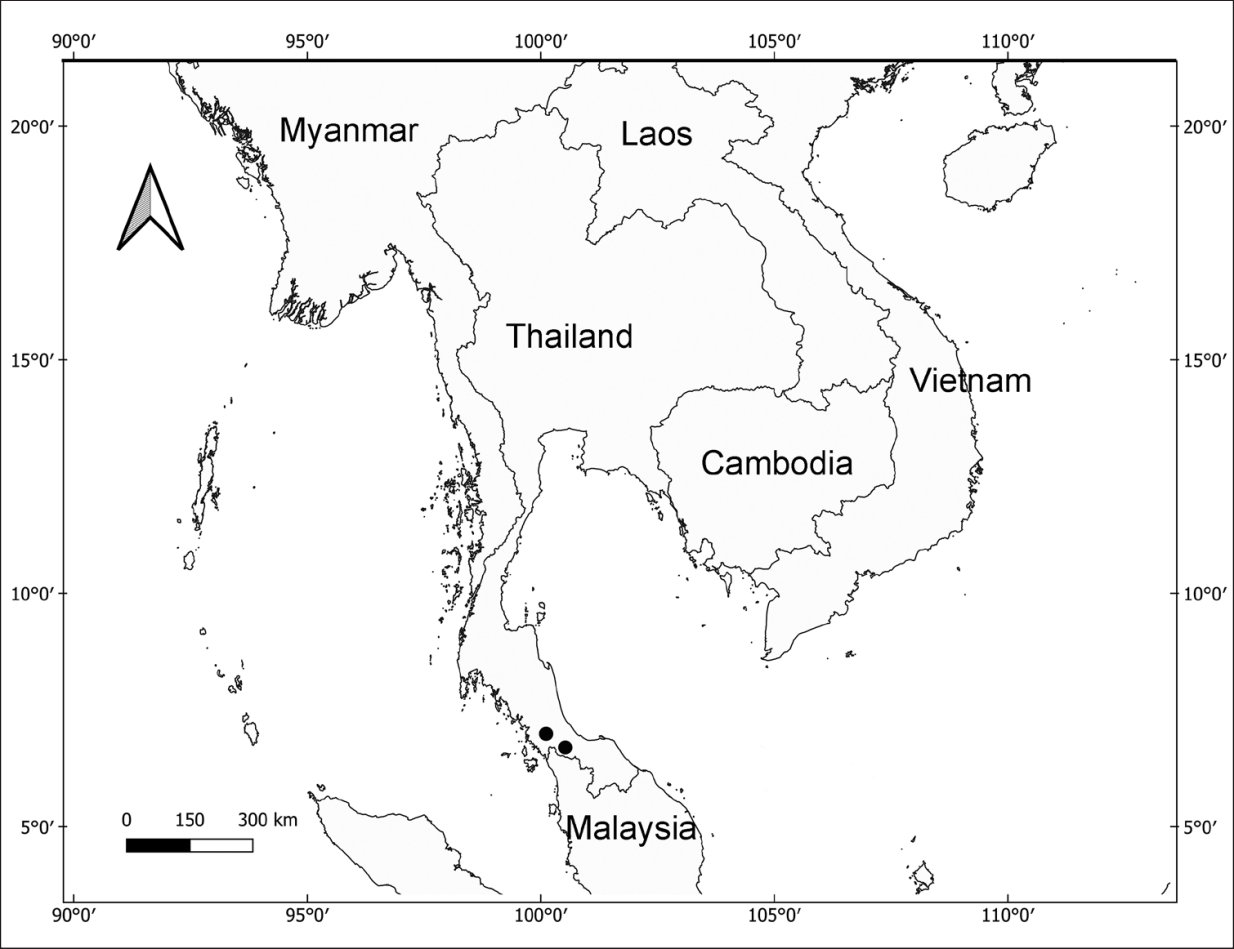
Distribution of *Aphyllorchisperiactinantha* A.Chantanaorr. & Chantanaorr. (black circle) in Thailand.

Field surveys were carried out in TNCW and Ban Yang Ko community forest, Songkhla province, southern Thailand (Fig. 1) in October 2017, November 2018, December 2020 and 2021. The specimens were photographed and deposited in BKF and PSU. Morphological characters were studied using an Olympus SZX12 stereomicroscope and the distinctive characters of the species were illustrated with the aid of an Olympus drawing tube (SZX-DA). The measurements and description were prepared from living plants and spirit materials. Comparisons of diagnostic characters were based on Thai specimens, as well as digital images of specimens held at BRI, BKF, K, PSU, and SING, available online, and on relevant taxonomic literature (e.g. Blume 1825; Downie 1925; Dockrill 1965; Seidenfaden 1978; Seidenfaden and Wood 1992; Hsieh et al. 2013; Tian et al. 2013; Pedersen 2014; Suetsugu et al. 2018; Qin et al. 2021). Herbarium acronyms follow Index Herbariorum (Thiers 2022). The preliminary conservation status was assessed following the International Union for Conservation of Nature (IUCN) Red List criteria (IUCN 2022) and using GeoCAT (Bachman et al. 2011) to calculate the area of occupancy (AOO) and extent of occurrence (EOO).

## ﻿Taxonomic treatment

### ﻿ Aphyllorchis periactinantha

Taxon classificationPlantaeAsparagalesOrchidaceae

A.Chantanaorr. & Chantanaorr. sp. nov.

31E74181-E7D4-596B-B410-A2B929B9262F

urn:lsid:ipni.org:names:77310465-1

Figs 2 , 3

#### Diagnosis.

Similar to *A.anomala*, but differs in having a concave labellum without purple veins, acute at the apex, and a semicircular rostellum.

#### Type.

Thailand. Songkhla Province: Ton Nga Chang wildlife sanctuary, ca. 100 m, 06°59'43.91"N, 100°09'00.57"E, 26 December 2020, *S. Chantanaorrapint & A. Chantanaorrapint 3109* (holotype: BKF!; isotype: PSU! [PSU00019495]).

#### Description.

Terrestrial, achlorophyllous herbs, with a suberect rhizome and an erect flowering shoot. ***Rhizomes*** pale brown, producing numerous horizontal roots; roots fleshy, 3.5–6.5 mm in diameter, pale brown, glabrous. ***Flowering shoots*** 70–150 cm tall, up to 9 mm in diameter at base, unbranched whitish or pale yellow, marked with purple stripes or spots, with 5–9 membranous sheaths at base and 10–15 scales above. ***Inflorescence*** racemose, terminal, 15–30 cm long, up to 32 flowers; rachis glabrous. ***Floral bracts*** reflexed, lanceolate to linear-lanceolate, acute to acuminate, 16.5–21.5 × 4.5–5.5 mm, whitish or pale yellow with purple stripes. ***Flowers*** opening widely, creamy white to pale yellow. ***Sepals*** creamy white to pale yellow, minutely tuberculate and sparsely pubescent on the adaxial surface, scattered with purple stripes or dots, concave, margins entire, apex acute; dorsal sepal narrowly ovate to lanceolate, 17.5–19.6 × 4.3–5.2 mm; lateral sepal obliquely narrow-ovate to obliquely lanceolate, 16.8–19.0 × 4.2–5.1 mm. ***Petals*** creamy white to pale yellow with purple veins, oblong-lanceolate, 16.0–16.5 × 3.9–4.3 mm, slightly falcate at apex, base obtuse to subtruncate, apex acute, margin entire or minutely erose and slightly recurved backward in the middle, keeled adaxially along midrib. ***Labellum*** creamy white to pale yellow, simple and not divided into hypochile and epichile, oblong-lanceolate, 16.2–17.0 × 3.8–4.5 mm, more or less folded along a midrib, concave at the basal part, apex acute, margin nearly entire or minutely erose, abaxial and adaxial surfaces nearly smooth. ***Column*** slender, subclavate, yellow at the apical third and purple at the proximal two thirds, gently curved throughout its entire length, 10–12 mm long, without column wing or a hook-shaped appendage on ventral side; clinandrium with a rather large central dome-like outgrowth; stigma more or less ovate in outline, shallowly bilobed at the lower margin; anther cap ovoid in outline, 2.0–2.2 mm long, apex obtuse; pollinia 2, soft and mealy, without caudicles. ***Pedicel with ovary*** slightly bent upwards or downwards, 18.5–23.5 mm long, ca. 2 mm in diameter, dark purple, pubescent, bearing sparse glandular hairs. ***Capsules*** (immature) pendulous, claviform or fusiform, 5.5–6.5 cm long, 0.7–1.1 cm diameter. ***Seeds*** not seen.

#### Additional specimens examined (paratypes).

Thailand. Songkla Province: Ton Nga Chang Wildlife Sanctuary, ca. 100 m, 06°59'43.91"N, 100°09'00.57"E, 22 October 2017, *S. Chantanaorrapint & A. Chantanaorrapint 2810* (PSU), 16 November 2018, *S. Chantanaorrapint & O. Suwanmala 2732* (PSU); Ban Yang Ko Community Forest, 7 December 2021, *C. Leeratiwong 21-1752* (PSU).

#### Phenology.

Flowering and fruiting observed from October to December.

#### Distribution, habitat and ecology.

*Aphyllorchisperiactinantha* is known only from two localities in Songkhla province (Fig. 1); however, it may also occur in other areas in southern Thailand with a similar vegetation type. The new species was found growing amongst leaf litter, in shade in lowland broad-leaf forest dominated by dipterocarps such as *Anisopteracostata* Korth., *Dipterocarpuskerrii* King, *Hopeaferrea* Laness., *H.odorata* Roxb., and *Shoreagratissima* (Wall. ex Kurz) Dyer, ca. 100 m above sea level. During the field surveys, we found a stingless bee (*Tetragonula* sp.) visiting the flower (Fig. 3D). However, its status as a pollinator could not be confirmed.

**Figure 2.**
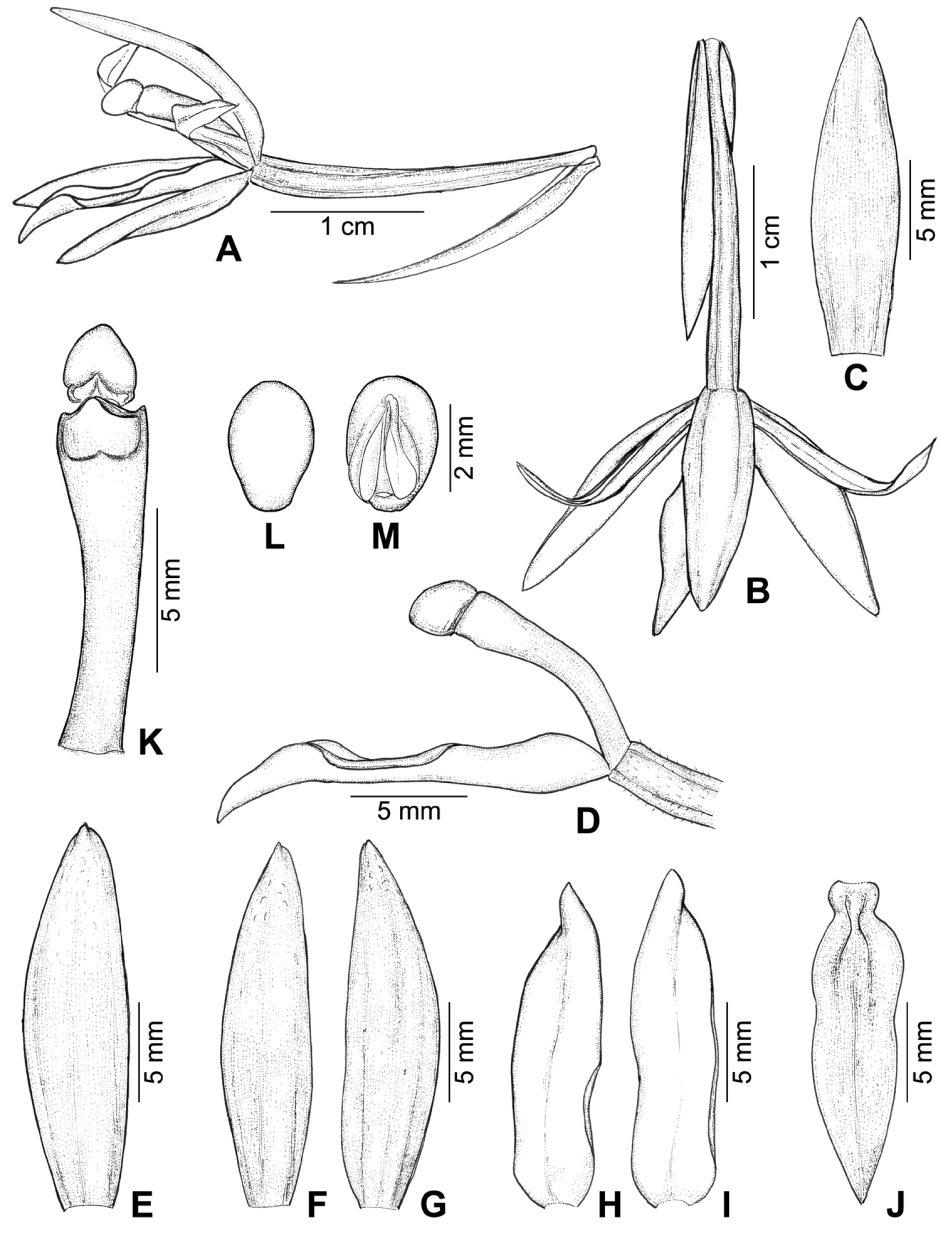
*Aphyllorchisperiactinantha* A.Chantanaorr. & Chantanaorr. **A** flower, lateral view **B** flower, top view **C** floral bract **D** column and labellum, side view **E** dorsal sepal **F, G** lateral sepals **H, I** petals **J** labellum **K** column and anther cap in ventral view **L, M** anther caps. Drawn by S. Chantanaorrapint.

**Figure 3.**
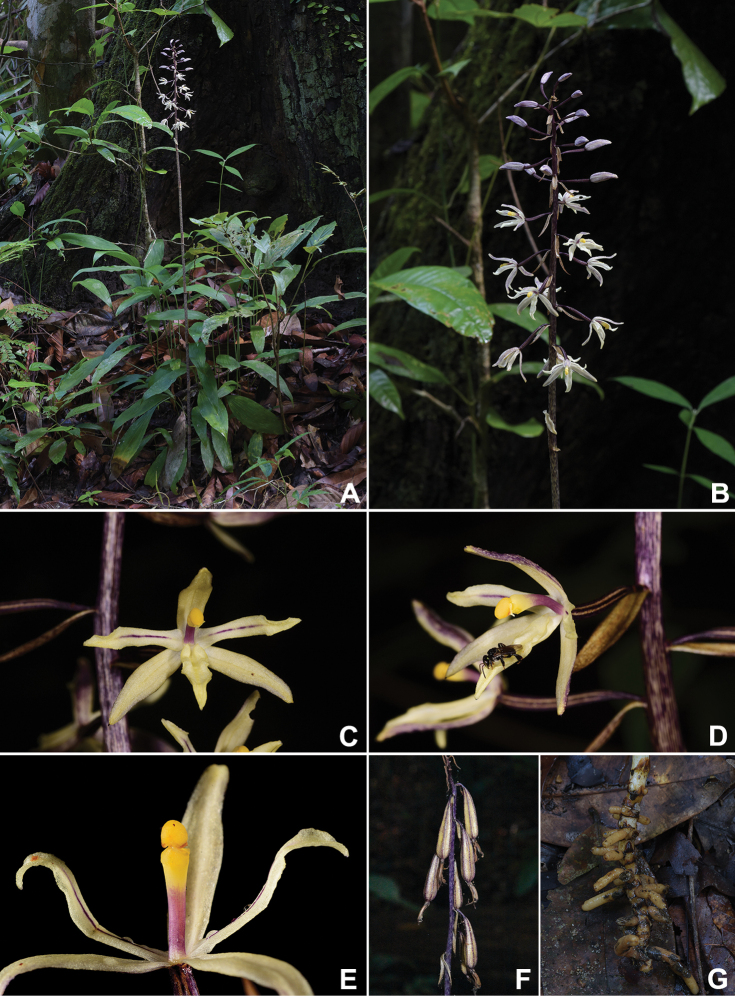
*Aphyllorchisperiactinantha* A.Chantanaorr. & Chantanaorr. *in situ***A** habit **B** inflorescence **C** flower in front view **D** ﬂower in oblique view with stingless bee (*Tetragonula* sp.) **E** column in ventral view **F** immature fruits **G** rhizome and roots **A–E**, **G** photographed by S. Chantanaorrapint **F** by C. Leeratiwong.

#### Etymology.

Greek prefix *peri*-, about, *actis*, ray or radiate, and *anthos*, flower, alluding to subactinomorphic flowers.

#### Conservation status.

This species is currently known from two subpopulations, representing two locations, which are in protected areas (Wildlife Sanctuary and community forest). One of the subpopulations is located beside a waterfall, which is a common visiting site for tourists. Therefore, habitat quality is threatened by trampling and other destructive activities potentially caused by regular visits by tourists to the area, namely the attraction of wild boars. Together, these have the potential to cause a population reduction. The other subpopulation is also somewhat disturbed by human activities such as illegal logging. Moreover, the number of mature individuals observed is fewer than 50. The extent of occurrence cannot be estimated because the species is known only from two subpopulations and its area of occupancy is estimated to be only 8 km^2^. *Aphyllorchisperiactinantha* is, therefore, assigned a preliminary status of Critically Endangered based on subcriterion C2a(i).

## ﻿Discussion

*Aphyllorchisperiactinantha* is morphologically similar to *A.anomala*, which is endemic to Queensland, Australia (Dockrill 1965). These two species share several common features, viz. the peloric flower, the simple labellum lacking any ridges or appendages and not being divided into hypochile and epichile, the wingless column, the clinandrium with a rather large central dome-like outgrowth, the bilobed stigma, and the ovoid anther cap.

The new species can be confused with *A.striata* from Peninsular Malaysia and Borneo (Seidenfaden and Wood 1992), in general appearance. However, *A.striata* differs from *A.periactinantha* in having smaller flowers, a trilobed labellum (with indistinct side lobes), an anther cap with an acute apex, and the lingulate rostellum.

The new species also resembles A.montanavar.rotundatipetala, endemic to Taiwan (Hsieh et al. 2013; Lin et al. 2016) and *A.simplex* from China and Vietnam in having peloric flowers and a simple labellum. However, A.montanavar.rotundatipetala can be easily distinguished from *A.periactinantha* by the obtuse apices of petals and labellum and the presence of staminodes on the ventral side of column. *Aphyllorchissimplex* differs from *A.periactinantha* by the shape of labellum and the apex of the column. The comparison of morphological characters between *A.periactinantha* and other related species is summarized in Table 1.

**Table 1.** Morphological differences among *Aphyllorchisperiactinantha*, *A.anomala*, A.montanavar.rotundatipetala, *A.simplex* and *A.striata*. The characters of previously described species are taken from the protologues and from recent publications (Ridley 1893; Dockrill 1965; Seidenfaden and Wood 1992; Averyanov 2011; Hsieh et al. 2013).

Characters	*A.periactinantha*	*A.anomala*	*A.montana var. rotundatipetala*	*A.simplex*	*A.striata*
dorsal sepal	narrowly ovate to lanceolate, 17.5–19.6 × 4.3–5.2 mm, apex acute	oblong-cuculate, ca. 12 × 3 mm, apex acuminate	oblong-cymbiform, 10–12 × 4.0–5.5 mm, apex obtuse	narrowly elliptic, 8–10 mm long, apex acute to obtuse	lanceolate, ca. 12–14 × 2.5–3.0 mm, apex acute
petals	oblong-lanceolate, 16.0–16.5 × 3.9–4.3 mm, apex acute	oblong, ca. 12 × 2 mm, apex acuminate	oblong, 9.5–11.5 × 3.5–4.0 mm, apex rotundate or obtuse	narrowly elliptic, 8–10 mm long, apex acute to obtuse	lanceolate, ca. 11–14 × 2.0–2.5 mm, apex acute
labellum	oblong-lanceolate, 16.2–17.0 × 3.8–4.5 mm, folded along a midrib, concave at the basal part apex acute, lateral margins not decurved	oblong-acuminate, ca. 11 × 2.5 mm, flat entirely, apex acuminate and slightly twisted, lateral margins decurved	oblong, 9.5–11.5 × 3.5–4.0 mm, flat entirely, apex obtuse or rotundate, lateral margins recurved backward at middle	oblanceolate, 9–10 × 2–2.5 mm, flat entirely, apex acute and slightly decurved, lateral margins slightly decurved	3-lobed with indistinct side lobes, ca. 11–14 × 2.5–3.0 mm, almost flat, apex acute, lateral margins rolled at the basal part
staminode	reduced	reduced	Present	present	reduced
rostellum	semicircular, entire apex	triangular, entire apex	ovate-lingulate, entire apex	ovate, emarginate apex	lingulate, entire apex

One could argue that the new species could simply represent an abnormal (peloric) form of another *Aphyllorchis* species, such as *A.maliauensis* Suetsugu, Suleiman & Tsukaya from Borneo (Suetsugu et al. 2018), which is overall similar in habit. However, these plants with peloric flowers have never been found growing in mixed populations with other *Aphyllorchis*. As this abnormality is constant, and as there is no definitive evidence indicating that it represents a peloric form of another known species, we feel it is justified to propose it as a new species.

There are now five species of *Aphyllorchis* known from Thailand. An updated key to distinguish these species is given below.

### ﻿Key to species of *Aphyllorchis* in Thailand

**Table:** 

1	Flowers subactinomorphic; labellum somewhat similar to the sepals, undivided into hypochile and epichile	***A.periactinantha***
–	Flowers zygomorphic; labellum distinctly different from the sepals, divided into hypochile and epichile	**2**
2	Sepals caudate, longer than 20 mm	**3**
–	Sepals rounded to obtuse, shorter than 20 mm	**4**
3	Hypochile with well-developed side lobes; epichile densely papillose on adaxial surface; anther apex rounded to obtuse	***A.caudata***
–	Hypochile with indistinct side lobes; epichile nearly glabrous on adaxial surface; anther apex forming a horn-like projection	***A.evrardii***
4	Sepals longer than 7 mm. Labellum longer than 6 mm; epichile ovate to slightly 3-lobed	***A.montana***
–	Sepals shorter than 7 mm. Labellum shorter than 6 mm; epichile cordate	***A.pallida***

## Supplementary Material

XML Treatment for Aphyllorchis periactinantha

## References

[B1] Averyanov LV (2011) The orchids of Vietnam illustrated surver Part 3. Subfamily Epidendroideae (primitive tribes-Neottieae, Vanilleae, Gastrodieae, Nervilieae). Turczaninowia 14(2): 15–100.

[B2] Bachman S Moat J Hill AW de la Torre J Scott B (2011) Supporting Red List threat assessments with GeoCAT: geospatial conservation assessment tool. In: Smith V Penev L (Eds) e-Infrastructures for data publishing in biodiversity science.ZooKeys 150: 117–126. 10.3897/zookeys.150.2109 PMC3234434 22207809

[B3] Blume CL (1825) *Aphyllorchis*. Tabellen en platen voor de Javaansche orchideën, Ter Lands, Batavia, t. 16.

[B4] Dockrill AW (1965) The genus *Aphyllorchis* Blume in Australia. Orchadian 1: 115–117. 10.5694/j.1326-5377.1965.tb71453.x

[B5] Downie DG (1925) Contributions to the flora of Siam. Bulletin of Miscellaneous Information 1925(10): 404–423. 10.2307/4115102

[B6] Hsieh S Leou C Yu S Lee C Yeh C (2013) *Aphyllorchisrotundatipetala* (Orchidaceae), a new species from Taiwan. Annales Botanici Fennici 50(3): 179–182. 10.5735/085.050.0309

[B7] IUCN (2022) Guidelines for Using the IUCN Red List Categories and Criteria. Version 15. https://www.iucnredlist.org/resources/redlistguidelines [accessed: 15.10.2022]

[B8] Lin TP Liu HY Hsieh CF Wang KH (2016) Complete list of the native orchids of Taiwan and their type information. Taiwania 61: 78–126. 10.6165/tai.2016.61.78

[B9] Pedersen HÆ (2014) *Aphyllorchis*. In: Santisuk T Balslev H (Eds) Flora of Thailand Vol.12(2). The Forest Herbarium, Department of National Parks, Wildlife and Plant Conservation, Bangkok, Thailand, 320–327.

[B10] POWO (2022) Plants of the World Online. Facilitated by the Royal Botanic Gardens, Kew. http://www.plantsoftheworldonline.org [accessed: 18.10.2022]

[B11] Pridgeon AM Cribb PJ Chase MW Rasmussen FN (2005) Genera Orchidacearum Volume 4 Epidendroideae (Part one). Oxford University Press, New York.

[B12] Qin Y Chen HL Deng ZH Liu Y (2021) *Aphyllorchisyachangensis* (Orchidaceae), a new holomycotrophic orchid from China. PhytoKeys 179: 91–97. 10.3897/phytokeys.179.63994 34285638 PMC8275566

[B13] Ridley HN (1893) On the flora of the Eastern coast of the Malay Peninsula. Transactions of the Linnean Society of London, 2^nd^ series: Botany 3: 267–408. 10.1111/j.1095-8339.1893.tb00678.x

[B14] Roy M Watthana S Stier A Richard F Vessabutr S Selosse AD (2009) Two mycoheterotrophic orchids from Thailand tropical dipterocarpacean forests associate with a broad diversity of ectomycorrhizal fungi. BMC Biology 7(1): 51. 10.1186/1741-7007-7-51 19682351 PMC2745373

[B15] Seidenfaden G (1978) Orchid genera in Thailand VI. Neottioideae Lindl. Dansk Botanisk Arkiv 32(2): 1–195.

[B16] Seidenfaden G Wood JJ (1992) The orchids of Peninsular Malaysia and Singapore. Olsen & Olsen, Fredensborg, 779 pp.

[B17] Suetsugu K Suleiman M Anthony F Tsukaya H (2018) *Aphyllorchismaliauensis* (Orchidaceae), a new species from the Maliau Basin, Sabah, Borneo. Phytotaxa 367(1): 85–90. 10.11646/phytotaxa.367.1.10

[B18] Thiers B (2022) [continuously updated] Index Herbariorum: A global directory of public herbaria and associated staff. New York Botanical Garden’s Virtual Herbarium. http://sweetgum.nybg.org/science/ih/ [accessed: 15 October 2022]

[B19] Tian H Li X Hu C (2013) A new synonym and lectotypification of *Aphyllorchiscaudata* (Orchidaceae). Kew Bulletin 68(2): 341–344. 10.1007/s12225-013-9451-3

